# Multiparametric MRI and transfer learning for predicting positive margins in breast-conserving surgery: a multi-center study

**DOI:** 10.1097/JS9.0000000000002278

**Published:** 2025-02-04

**Authors:** Xue Zhao, Jing-Wen Bai, Sen Jiang, Zhen-Hui Li, Jie-Zhou He, Zhi-Cheng Du, Xue-Qi Fan, Shao-Zi Li, Guo-Jun Zhang

**Affiliations:** a Fujian Key Laboratory of Precision Diagnosis and Treatment in Breast Cancer & Xiamen Key Laboratory of Endocrine-Related Cancer Precision Medicine, Xiang’an Hospital of Xiamen University, School of Medicine, Xiamen University, Xiamen, China,; b National Institute for Data Science in Health and Medicine, Xiamen University, Xiamen, China,; c The Breast Center and the Cancer Institute, Yunnan Cancer Hospital & The Third Affiliated Hospital of Kunming Medical University & Peking University Cancer Hospital, Kunming, China,; d Department of Radiology, Cancer Hospital of Shantou University Medical College, Shantou, China,; e Department of Radiology, Yunnan Cancer Hospital & The Third Affiliated Hospital of Kunming Medical University & Peking University Cancer Hospital Yunnan, Kunming, China,Xiang’an Hospital of Xiamen University, School of Medicine, Xiamen University, Xiamen, China; f Institute of Artificial Intelligence, Xiamen University, Xiamen, China and; g Department of Artificial Intelligence, Xiamen University, Xiamen, China

**Keywords:** breast-conserving surgery, multiparameter magnetic resonance imaging, surgical margins, transfer learning

## Abstract

This study aimed to predict positive surgical margins in breast-conserving surgery (BCS) using multiparametric MRI (mpMRI) and radiomics. A retrospective analysis was conducted on data from 444 BCS patients from three Chinese hospitals between 2019 and 2024, divided into four cohorts and five datasets. Radiomics features from preoperative mpMRI, along with clinicopathological data, were extracted and selected using statistical methods and LASSO logistic regression. Eight machine learning classifiers, integrated with a transfer learning (TL) method, were applied to enhance model generalization. The model achieved an AUC of 0.889 in the internal test set and 0.771 in the validation set. Notably, TL significantly improved performance in two external validation sets, increasing the AUC from 0.533 to 0.902 in XAH and from 0.359 to 0.855 in YNCH. These findings highlight the potential of combining mpMRI and TL to provide accurate predictions for positive surgical margins in BCS, with promising implications for broader clinical application across multiple hospitals.

## Introduction

Breast cancer is the most common malignancy and a leading cause of cancer-related deaths in women globally. Breast-conserving surgery (BCS), followed by radiotherapy, is the standard treatment for early-stage breast cancer, offering survival outcomes comparable to mastectomy while preserving cosmetic appearance[1]. However, positive surgical margins, defined as the presence of tumor cells at or near the resection edge, remain a critical risk factor for local recurrence, even with adjuvant treatments like radiotherapy[2]. Reported positive margin rates range from 6.8% to 23.9%^[3,4]^, with individual surgeon rates varying from 7.8% to 36.8%[4], reflecting substantial variability across different clinical centers and surgeons. Despite extensive research, predicting positive margins using clinicopathological factors alone has demonstrated limited predictive accuracy[5]. Magnetic resonance imaging (MRI) is valuable for detecting early-stage breast cancer and preoperatively identifying tumor location, multi-centricity, and potential positive margins[6]. Radiomics, which extracts high-dimensional features from medical images, has emerged as a promising tool for characterizing breast cancer and predicting outcomes. However, its application in predicting positive surgical margins remains underexplored. Additionally, transfer learning (TL) offers a potential solution for addressing variability in imaging protocols by enabling models trained on one dataset to perform effectively on diverse external datasets[7]. The aim of this study is to develop an MRI-based radiomics model that combines MRI features with clinicopathological data to predict the risk of positive surgical margins in BCS, ensuring generalizability across different clinical centers.

## Material and methods

In this retrospective multicenter study, data from 444 BCS patients across three Chinese hospitals (2019–2024) were analyzed to develop a predictive model for positive surgical margins. The inclusion criteria encompassed female patients aged 18–75 with histologically confirmed breast cancer who underwent BCS and had preoperative MRI within one month prior to surgery. Patients were divided into five datasets: a training set, an internal test set, a validation set, and two external validation sets. Preoperative breast MRI was conducted using 1.5 T or 3.0 T scanners, and radiomic features were extracted from DCE-MRI, T2WI, and DWI sequences using PyRadiomics. Feature selection was performed using *t*-tests and LASSO regression. Eight machine learning classifiers were tested, with model performance evaluated using area under the receiver operating characteristic curve (AUC), accuracy, sensitivity, and specificity. TL was incorporated to improve the model’s generalizability across external centers. The models were compared against clinicopathological and radiomics-only models. Detailed methodologies and the study design flowchart can be found in Supplementary Data, http://links.lww.com/JS9/D863.

## Results

### Patient characteristics

**Figure 1.**
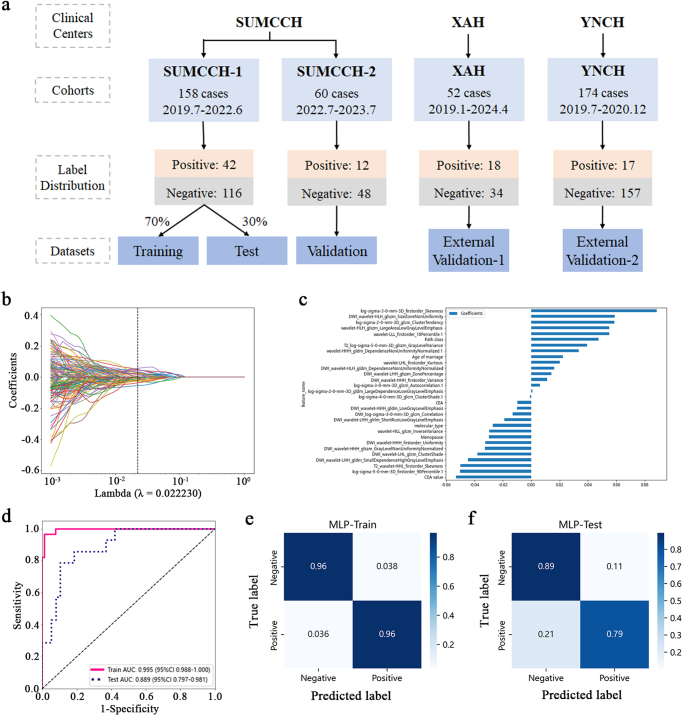
The flowchart of patient enrollment and the performance of Base-MMRM. (A) Patients from center 1 were divided into SUMCCH-1 and SUMCCH-2. The SUMCCH-1 cohort was randomly split into the training (70%) and test sets (30%). SUMCCH-2 was assigned as a validation cohort, and patients from centers 2 and 3 were assigned as two external validation cohorts. (B) LASSO coefficient profiles of the radiomics features compared to the lambda values. The vertical line of the optimal lambda was drawn according to the ten-fold cross-validation. (C) The selected radiomics features (with nonzero coefficients) and their coefficients. (D) The ROC curve of MMRM in training and test sets. (E) The confusion matrix of MMRM in the training set. (F) The confusion matrix of the MMRM in the test set.

A total of four patient cohorts from SUMCCH, XAH, and YNCH in Guangdong, Fujian, and Yunnan Provinces were included (Fig. 1a). The SUMCCH-1 cohort (158 patients) was divided into a training set (106 cases) and an internal test set (52 cases), with no significant differences in clinicopathological features between the two sets. The SUMCCH-2 cohort (60 cases), a validation set, had no significant differences from the training set. Two additional external validation sets from XAH (52 cases) and YNCH (174 cases) showed significant differences, particularly in the YNCH cohort, where 7 features had notable disparities. The positive margin rates were 26.6%, 20.0%, 34.6%, and 9.8% in SUMCCH-1, SUMCCH-2, XAH, and YNCH, respectively (Table S1 in Supplementary Data. http://links.lww.com/JS9/D800).

### Predictive models using clinicopathological and radiomic features

Single-parameter models were developed using clinicopathological data and MRI features from DCE-MIP, T2WI, and DWI sequences to predict positive surgical margins (Table S2 in Supplementary Data. http://links.lww.com/JS9/D800). Among these, the DCE-MIP radiomics model achieved the highest performance with an AUC of 0.688 and an accuracy of 0.692 in the internal test set. Models based on clinicopathological features alone also performed comparably, with an AUC of 0.684. Radiomic features extracted from both the tumor and peritumoral regions showed similar predictive capability, with the 2 mm peritumoral margin features performing best (AUC 0.681).

### Multi-parameter models for enhanced performance

**Figure 2.**
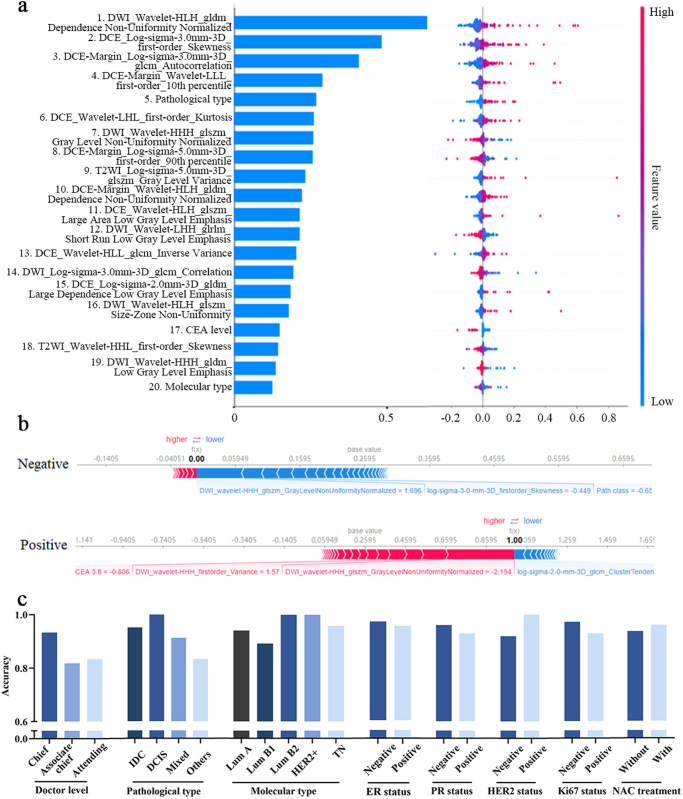
Model’s visualization and subgroup analysis of Base-MMRM in the SUMCCH-1 cohort. (A) Variance importance plot (left) calculated by SHapley Additive exPlanations (SHAP), listing the most significant variables in descending order. Summary plot (right) calculated by SHAP, representing the feature impacts on the decision of the MMRM and interaction between radiomic features in MMRM. (B) Force plot of representative cases of patients with negative or positive surgical margins in BCS. (C) Subgroup analysis of the accuracy of Base-MMRM predictions in SUMCCH-1. IDC, infiltrating ductal carcinoma; DCIS, ductal carcinoma in situ; Lum, luminal; HER2, human epidermal growth factor receptor 2; HER2 +, HER2 overexpression; TN, triple negative; ER, estrogen receptor; PR, progesterone receptor; NAC, neoadjuvant chemotherapy. The symbol * represents *P* ≤ 0.05.

To improve prediction accuracy, multi-parameter models integrating clinicopathological data with radiomic features were developed (Table S3 in Supplementary Data. http://links.lww.com/JS9/D800). A comprehensive model combining tumor and peritumoral features from DCE-MIP, T2WI, DWI, and clinicopathological data outperformed the best single-parameter model. The optimized comprehensive multi-parameter model was named Base-MMRM. 30 critical features were selected using the *t*-test and LASSO regression (Fig. 1b-c and Table S4 in Supplementary Data. http://links.lww.com/JS9/D800). Base-MMRM demonstrated excellent performance, achieving an AUC of 0.995 in the training set and 0.889 in the test set (Fig. 1d), with a sensitivity of 0.79 and a specificity of 0.89 (Fig. 1e-f). SHAP analysis highlighted key predictive features, including DCE-MIP first-order skewness and DWI wavelet features (Fig. 2a-b). The model maintained high accuracy across different patient subgroups (Fig. 2c), underscoring its robustness and applicability.

### External validation and transfer learning

**Figure 3.**
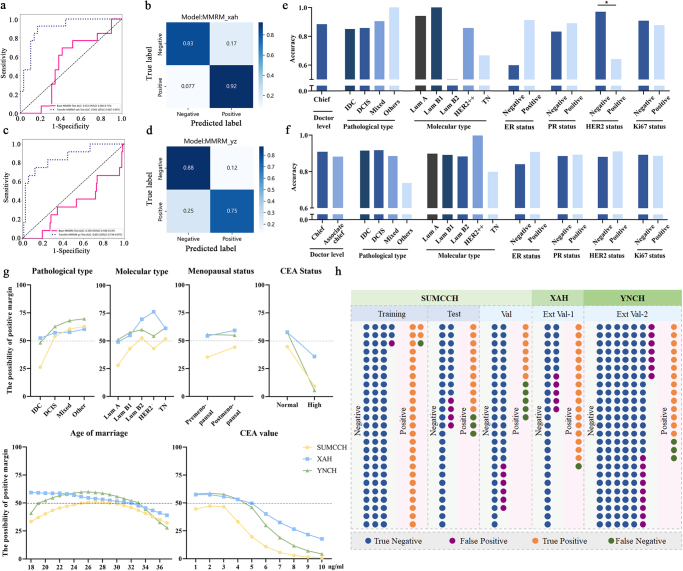
The performance and visualization of Transfer-MMRMs in external centers and prediction analysis profiles of MMRMs. (A) The ROC curve of the Base-MMRM and Transfer-MMRM-XAH in the XAH cohort. (B) The confusion matrix of the Transfer-MMRM-XAH in the XAH cohort. (C) The ROC curve of Base-MMRM and Transfer-MMRM-YNCH in the YNCH cohort. (D). The confusion matrix of Transfer-MMRM-YNCH in the YNCH cohort. (E). Subgroup analysis of the accuracy of Transfer-MMRM-XAH predictions in XAH. (F) Subgroup analysis of the accuracy of Transfer-MMRM-YNCH predictions in YNCH. (G) The analysis of the impact of each clinicopathological feature on the risk of positive margins predicted by the MMRMs in three clinical centers. (H) The number of true-negative, false-negative, true-positive, and false-positive events for the MMRMs in each cohort. IDC, infiltrating ductal carcinoma; DCIS, ductal carcinoma in situ; Lum, luminal; HER2, human epidermal growth factor receptor 2; HER2 +, HER2 overexpression; TN, triple negative; ER, estrogen receptor; PR, progesterone receptor. Val, validation; Ext: external. The symbol * represents *P* ≤ 0.05.

Base-MMRM was validated on the SUMCCH-2 cohort, achieving an AUC of 0.771 and an accuracy of 0.817. TL was applied to improve performance on external validation sets. The Transfer-MMRM-XAH model demonstrated an AUC of 0.902 and an accuracy of 0.857 (Fig. 3a-b), while the Transfer-MMRM-YNCH model achieved an AUC of 0.855 and an accuracy of 0.864 (Fig. 3c-d). Subgroup analysis confirmed the strong generalizability of Transfer-MMRMs across different patient subtypes (Fig. 3e-f). Additionally, simulated data were used to evaluate the influence of clinicopathological features on predictions across the three centers using MMRMs. The analysis revealed that invasive ductal carcinoma (IDC) patients had lower risks of positive margins, whereas patients with luminal B (HER2 +) and triple-negative breast cancer exhibited higher risks (Fig. 3g). A comprehensive visualization diagram summarizing the predictions of MMRMs is presented in Figure 3h.

In conclusion, the Base-MMRM demonstrated robust performance in internal validation, while Transfer-MMRMs significantly improved generalization to external cohorts, showing its potential for accurately predicting positive surgical margins in BCS across diverse clinical settings.

## Discussion

This study highlights the potential of mpMRI radiomics profiles and TL in predicting positive surgical margins in BCS across multiple clinical centers. Unlike the previous model relying on a single MRI parameter (DCE) for predicting surgical margins in HER2 + breast cancer[8], our study employed a comprehensive mpMRI dataset (DCE, T2WI, and DWI) integrated with clinicopathological features. Besides, our Base-MMRM incorporated an additional DWI series than the CSS model developed by Ma *et al.* and achieved a higher AUC (0.889 vs. 0.79) in the test set[9].

Challenges such as multicenter variability in imaging protocols were addressed using TL via weight initialization, improving model generalization without compromising patient data privacy. This approach mitigated inter-center disparities while ensuring robust performance across diverse datasets. Key features identified included IDC type, HER2 status, and specific tumor subtypes, emphasizing the model’s ability to inform personalized surgical strategies.

While TL showed promise, the study acknowledges several limitations, including potential selection bias due to the small sample size and the exclusion of other imaging modalities like ultrasound. Future studies with larger, prospective, multicenter cohorts are essential to validate these findings for broader clinical application. Moreover, the exploration of more advanced and optimized models, such as deep learning and ensemble approaches[10], is recommended to further improve performance.

## Conclusion

In conclusion, MMRMs offer a promising, non-invasive method for preoperative assessment of surgical margins in BCS. The application of TL further enhances the generalizability and predictive accuracy of the models across diverse clinical settings. This research supports the development of intelligent decision-making tools for personalized surgical interventions, with significant clinical implications for improving the safety and efficacy of BCS.

## Data Availability

The data included in the study are available from the corresponding author upon reasonable request.

## Research registration unique identifying number (UIN)

This study was registered in the Chinese Clinical Trial Registry (Registration No. ChiCTR2400083298).
